# Optimizing lazertinib therapy through GSTM1 genotyping: a strategy to reduce excess drug exposure and potential toxicity

**DOI:** 10.1007/s00280-025-04828-y

**Published:** 2025-11-07

**Authors:** Rob ter Heine, Bianca J. C. van den Bosch, Robin M. van Geel, Wouter H. van Geffen, Lizza E. L. Hendriks, Michel M. van den Heuvel, Simon E. Koele, Adrianus J. de Langen, Thijs H. Oude Munnink, Anthonie J. van der Wekken

**Affiliations:** 1 Department of Pharmacy, Pharmacology & Toxicology Radboudumc, Geert Grooteplein Zuid 10, 6525GA Nijmegen, The Netherlands; 2 Department of Clinical Genetics, Clinical Genomics, Maastricht University Medical Center, Maastricht, The Netherlands; 3 Department of Clinical Pharmacy & Toxicology, Maastricht University Medical Center, Maastricht, The Netherlands; 4 Department of Pulmonology, Radboudumc, Nijmegen, The Netherlands; 5 Department of Pulmonary Diseases, Maastricht University Medical Center, Maastricht, The Netherlands; 6 Department of Pulmonology, University Medical Center Utrecht, Utrecht, The Netherlands; 7 Department of Thoracic Oncology, Netherlands Cancer Institute, Amsterdam, The Netherlands; 8 Department of Clinical Pharmacy and Pharmacology, University Medical Center Groningen, Groningen, The Netherlands; 9 Department of Pulmonary Diseases, University of Groningen, University Medical Centre Groningen, Groningen, The Netherlands

**Keywords:** Lazertinib, Pharmacogenetics, Pharmacogenomics, Dose individualization, EGFR

## Abstract

**Purpose:**

The combination of lazertinib and amivantamab has shown superior efficacy over first line osimertinib in EGFR-mutated metastatic non-small cell lung cancer, but is associated with significant toxicity and high costs. Lazertinib exposure varies widely due to genetic polymorphisms of the encoding for *GSTM1*, with almost 50% of Caucasians having a non-functional enzyme resulting in an approximate twofold higher systemic drug exposure. Despite this, all patients receive a fixed 240 mg once-daily dose irrespective of *GSTM1* status, leading to avoidable toxicity without additional clinical benefit. Our purpose was to develop alternative dosing regimens based on GSTM1 status.

**Methods:**

We conducted pharmacokinetic simulations using an existing validated population pharmacokinetic model to evaluate genotype-guided alternative dosing strategies in *GSTM1* null individuals.

**Results:**

Two regimens— 160 mg once daily (QD) and 240 mg every other day—were predicted to provide systemic exposures comparable to or exceeding those seen in *GSTM1* non-null patients on the standard dose. The 160 mg QD dose resulted in a geometric mean ratio in GSTM1 null patients (GMR) for the trough (Ctrough) and average (Caverage) concentration relative tot he approved dose in *GSTM1* non-null patients of 1.43 and 1.19, respectively. The respective GMRs for Ctrough and Caverage associated with 240 mg every-other-day dosing were 0.90 and 0.89, and this dosing regimen could reduce drug expenses up to 50% ($132.860 per year per patient) based on current pricing.

**Conclusion:**

Our findings support the feasibility of individualized lazertinib dosing based on *GSTM1* status to reduce toxicity and healthcare costs without compromising effective exposure.

## Introduction

In the pivotal phase III MARIPOSA trial, the combination of lazertinib and amivantamab improved overall survival (OS) for previously untreated epidermal growth factor receptor (EGFR, exon 19 deletion or exon 21 L858R point mutation)-mutated metastatic non-small cell lung cancer (NSCLC) when compared to osimertinib monotherapy. These findings led to the regulatory approval of this combination and the incorporation in clinical guidelines [1–3].

Despite the high efficacy of this treatment, it is associated with considerable toxicity. In the MARIPOSA trial, grade 3 adverse events occurred in 75% of the patients in the amivantamab-lazertinib group, leading to treatment interruptions and treatment discontinuation of this combination therapy in over 80% and 30% of the patients, respectively, which is in part caused by lazertinib [1]. The optimal individual dose for lazertinib is currently unknown. In the approved one-dose-fits all regimen at 240 mg once daily (QD), the level of systemic lazertinib exposure in monotherapy or in combination with amivantamab is not associated with efficacy [4, 5]. This may be explained by the fact that in the approved dose the majority of patients has a systemic exposure that is well above the proposed efficacy threshold trough concentration of 0.0568 mg/L [4]. This implies that, like other EGFR-inhibitors, lazertinib is dosed high in the plateau of the dose–response curve [6]. Nonetheless, in the approved dosing regimen, lazertinib exposure is significantly correlated with EGFR-inhibitor associated toxicity, like rash, paresthesia and stomatitis [4, 5]. The clinical benefit of the amivantamab–lazertinib combination is, therefore, blunted by its pronounced toxicity profile, particularly in vulnerable and frail populations. Furthermore, with the full-dose combination of amivantamab and lazertinib costing approximately $500.000 to $600.000 per patient per year [7], this treatment puts a serious strain on already limited healthcare budgets.

Inter-individual pharmacokinetic variability of lazertinib is high, caused by a genetic polymorphism in the gene encoding for the enzyme glutathione S-transferase Mu 1 (*GSTM1*) [4, 5]. This enzyme is responsible for the metabolism of lazertinib (but not amivantamab) and in the Caucasian population, approximately 50% of the patients have a non-functional enzyme (*GSTM1* null genotype) [8]. This null genotype results in a two-fold higher systemic lazertinib exposure compared to patients with a functional enzyme [4, 5]. According to the label, each patient receives the same dose, irrespective of the *GSTM1* genotype. Therefore, half of all patients receives a higher-than-necessary dose of lazertinib—exposing them to added toxicity without clinical benefit. This is underlined by the fact that progression-free survival, the primary endpoint of the MARIPOSA study, was not associated with the *GSTM1* genotype [4, 5].

Since *GSTM1* genotyping is often already routinely available using standard real-time polymerase chain reaction assays [9], it offers a practical approach to individualize dosing and reduce unnecessary toxicity without compromising effective exposure. Therefore, our aim was to explore alternative dosing regimens that would result in reduced costs and/or toxicity without compromising effective exposure by means of pharmacokinetic modelling.

## Methods

We conducted a pharmacokinetic (PK) simulation study utilizing the population PK model developed by the license holder, as detailed in regulatory submissions to the U.S. Food and Drug Administration (FDA) and European Medicines Agency (EMA) [4, 5]. This model, which was constructed using data from 1,389 individuals from various ethnic backgrounds enrolled in the clinical development program for lazertinib, and includes the *GSTM1* genotype as a covariate for drug clearance. In the dataset used for this population pharmacokinetic analysis, the median body weight was 63.1 kg, with a standard deviation of 14.2 kg. In the final model *GSTM1* status, weight, sex, ethnicity (Japanese versus non-Japanese) and pretreatment (treatment-naive versus not treatment-naive) were the included covariates.

Using this model, we simulated the PK profiles of various dosing regimens incorporating the currently available 80 mg and 240 mg tablet strengths. The evaluated regimens included the approved dose of 240 mg once daily (QD) irrespective of genotype, as well as two genotype-guided alternative regimens for individuals with the GSTM1 null genotype: 160 mg QD and 240 mg every other day. While once-daily dosing is generally favoured to support treatment adherence, the every-other-day dosing regimen was considered due to significant cost implications. Specifically, the cost of a daily 160 mg dose (administered as two 80 mg tablets) is the same as a 240 mg dose, at $728 per day based on Micromedex Red Book data in June 2025 [7].

Pharmacokinetic simulations were performed to predict the steady-state plasma concentrations of lazertinib for a reference individual (70 kg, non-Japanese treatment-naïve male) under each dosing regimen. Additionally, Monte Carlo simulations were conducted in 1,000 virtual representative non-Japanese treatment-naïve patients per regimen. We assumed a total body weight of 70 kg with a inter- individual variability of 20% (log-normal coefficient of variation) representative for a European population [10], with a distribution of 1:1 of women and men. We used this simulation to derive the predicted steady-state trough concentration (C_trough_) and average weekly concentration (C_average_) as primary endpoints of our analysis. An alternative dosing regimen for patients with the *GSTM1* null genotype was considered acceptable if the geometric means of the associated C_trough_ and C_average_ were not > 20% lower than those associated with the approved lazertinib dose in patients with the *GSTM1* non-null genotype. This criterion was derived from FDA and EMA bioequivalence guidelines, which state that a generic drug can be considered equally effective and safe based on pharmacokinetic endpoints if the geometric mean ratios for these pharmacokinetic endpoints are within 0.8–1.25 [11, 12]. As an exploratory secondary endpoint, the fraction of patients above the earlier proposed therapeutic threshold of 0.0568 mg/L for the C_trough_ at pharmacokinetic steady-state was calculated from the Monte Carlo simulation results. Finally, the potential maximum yearly cost savings associated with the genotype-guided alternative regimens were assessed based on publicly available drug pricing information [7] under the assumption of administration of a full dose during a year in the United States.

## Results

**Fig. 1:**
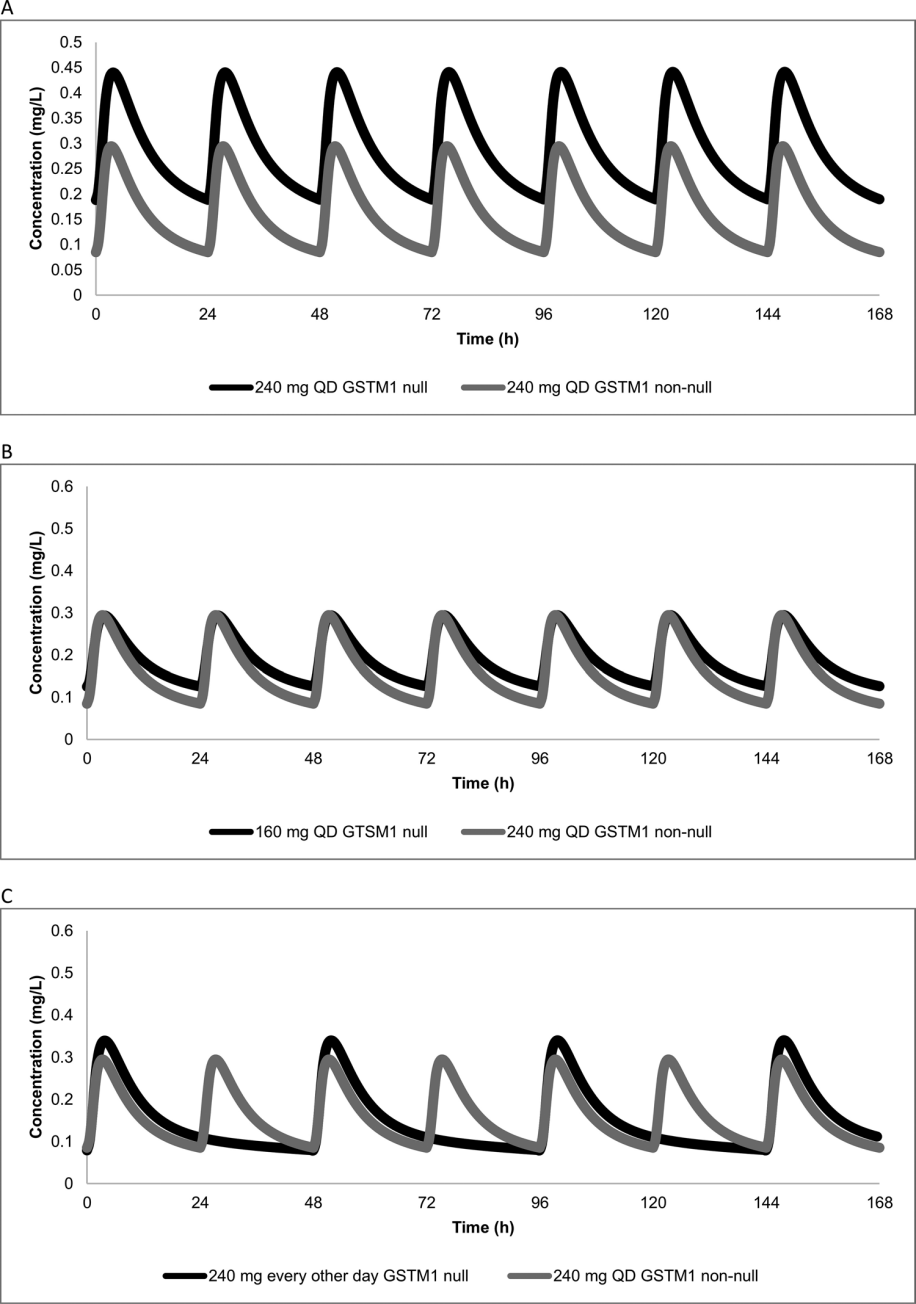
Panel A shows the predicted steady-state lazertinib pharmacokinetics for approved 240 mg dose for a typical individual with a *GSTM1* null and a *GSTM1* non-null genotype. Panel B and C show the predicted pharmacokinetics for the 160 mg QD and the 240 mg every other day dosing regimens for a typical individual with the *GSTM1* null genotype, together with the predicted pharmacokinetics for an individual with the *GSTM1* non-null genotype receiving the approved dose as a reference

**Fig. 2:**
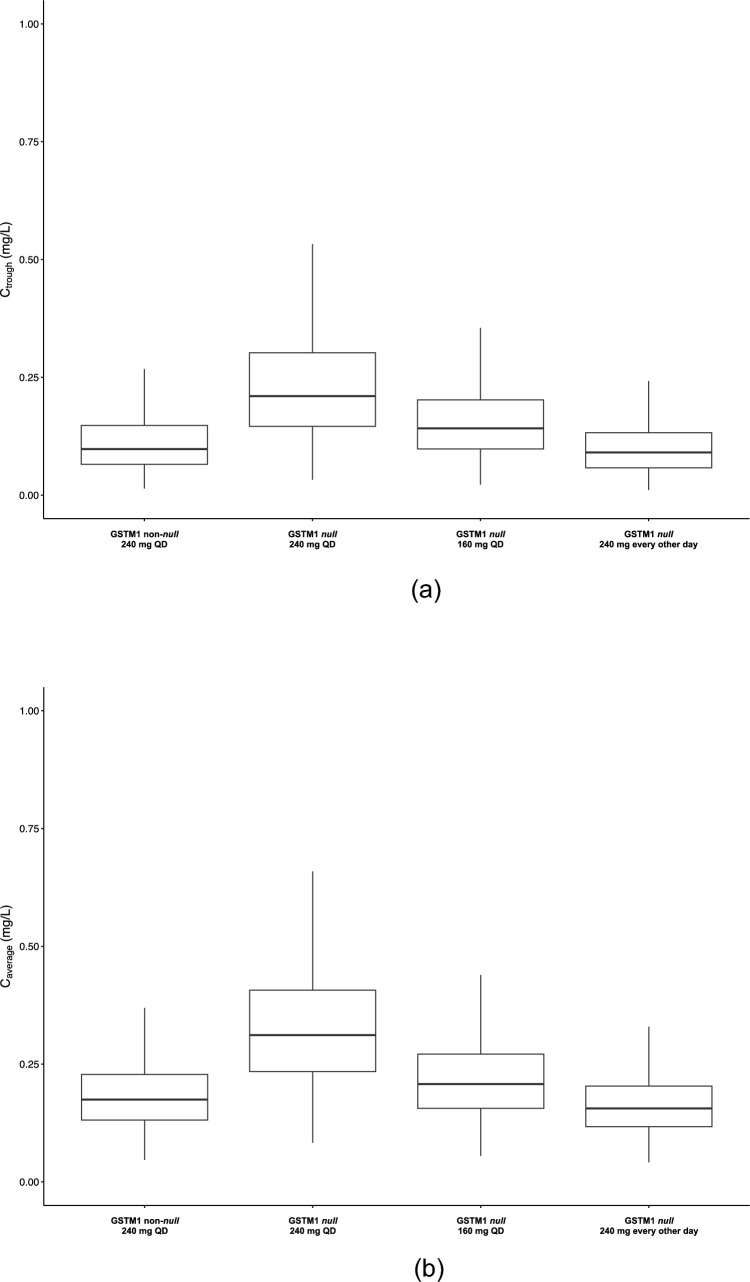
Panel A shows the Box and Whiskers plot for the lazertinib C_trough_ for the various dosing regimens and genotypes at pharmacokinetic steady-state during a week. Panel B shows the Box and Whiskers plot for the lazertinib C_average_ for the various dosing regimens during a week at pharmacokinetic steady-state. The box depicts the predicted interquartile range; the solid horizontal line depicts the median and the whiskers depict the minimum and maximum

The results of the pharmacokinetic simulations are graphically summarized in Fig. 1, showing the steady-state pharmacokinetics of the various dosing regimens and genotypes during a week at steady state for a typical male treatment-naïve patient weighting 70 kg. Figure 2 depicts Box and Whisker plots of the predicted Ctrough and Caverage for the various scenarios. Table 1 shows the results of the Monte Carlo simulation.

**Table 1:** Monte Carlo simulation results for steady-state lazertinib pharmacokinetics

	*GSTM1* non-null Approved dose	*GSTM1* null Approved dose	*GSTM1* null Alternative dose	
	240 mg QD	240 mg QD	160 mg QD	240 mg every other day
Geometric mean Ctrough (%CV)	0.0962 mg/L (64.1%)	0.206 mg/L (56.2%)	0.138 mg/L (56.0%)	0.0864 mg/L (64.0%)
Geometric mean ratio for Ctrough compared to approved dosing regimen in GSTM1 non-*null* genotype			1.43	0.90
Geometric mean Caverage (%CV)	0.174 mg/L (42.5%)	0.310 mg/L (42.5%)	0.207 mg/L (42.5%)	0.155 mg/L (42.5%)
Geometric mean ratio Caverage compared to approved dosing regimen in GSTM1 non-*null* genotype			1.19	0.89

CV: coefficient of variation, C_trough_: trough concentration, C_average_: average concentration

As expected, the approved dose of 240 mg QD was associated with an approximate two-fold increased Ctrough and Caverage in the population with *GSTM1* null genotype, compared to the population with the non-null genotype (geometric mean ratios 2.14 and 1.78). Furthermore, none of the genotype-guided alternative reduced dosing regimens in patients with the *GSTM1* null genotype was predicted to result in relevantly lower exposure than the approved 240 mg QD dose for the *GSTM1* non-null genotype, as the geometric mean ratios were > 0.8 for all pharmacokinetic endpoints (Table 1). Therefore, these alternative dosing regimen predictions are considered equivalent. The fraction of patients with a Ctrough at steady-state above the proposed efficacy treshold of 0.0568 mg/L for patients with the *GSTM1* non-null genotype at the approved dose (reference dose) and patients with the *GSTM1* null genotype for the 240 mg every other day and the 160 mg once daily dose (alternative dose) are respectively 81.7%, 76.3% and 94.7%.

The dosing regimen of 240 every other day is expected to result in a maximum annual cost reduction of 50% per patient for lazertinib. Since the approved dose costs $728 per day [7], the yearly costs of the full daily dose are $265.720 and a dose reduction to every other day dosing results in a reduction of drug expenses of $132.860, while no reduction in costs was anticipated for the 160 mg QD regimen, as the 160 mg and 240 mg tablets are priced identically.

## Discussion

Genotype-guided dose adjustments of lazertinib in patients with the *GSTM1* null genotype are predicted to result in systemic exposure that is equivalent to or higher than the established safe and efficacious exposure associated with the 240 mg once-daily regimen in patients with *GSTM1* non-null genotype. The predicted slight difference systemic exposure would be accepted in a formal bioequivalence study for a generic Lazertinib formulation, in line with regulatory guidelines [11, 12]. Therefore, the proposed alternative dosing regimens are not expected to compromise therapeutic efficacy. Furthermore, given the known positive correlation between systemic exposure to lazertinib monotherapy and the incidence of adverse effects [5], we expect that such a reduction in exposure through genotype- based dosing likely leads to a lower probability of toxicity in *GSTM1* null patients. In addition, the 240 mg every-other-day regimen is anticipated to significantly reduce overall drug costs. As expected, the predicted fraction above the postulated threshold for Ctrough at steady-state above of 0.0568 mg/L for patients with *GSTM1* null genotype with alternative dosing regimens was similar or higher than the fraction predicted for the reference population (*GSTM1* non-null with a dose of 240 mg QD).

With the *GSTM1* null genotype being present in approximately 50% of the global population [8], the implementation of personalized dosing strategies based on *GSTM1* status has the potential to confer substantial benefits not only at the individual patient level, but also at the population level. Since *GSTM1* genotype impacts the clearance of lazertinib by an approximate two-fold and the inter-individual variability in clearance is approximately 37%, this shows that the impact of *GSTM1* on drug exposure is not negligible and that genotyping can be used to individualize the dose [5]. A prerequisite to implement a genotype-based dosing strategy is an acceptable turn-around time (e.g. 5 working days) for *GSTM1* genotyping, which has previously been proven feasible [13].

Considering the relatively low cost of *GSTM1* genotyping in the order of magnitude of several hundreds of dollars [14] and the high treatment costs of lazertinib and its associated adverse event management [15], this approach may represent a cost-effective strategy for optimizing treatment outcomes in this patient subgroup, considering the potential maximal savings of $132.860 per person per year. In our analysis, pragmatically the maximal annual savings were calculated. In the MARIPOSA trial [1], the median progression-free survival was 23.7 months, which indicates that potential total savings in *GSTM1* null patients might be even higher.

From a pharmacoeconomic standpoint, the alternative dosing regimen of every other day is preferable. Nonetheless, this intermittent schedule introduces additional complexity compared to a once-daily regimen and may consequently compromise patient adherence [16]. For individuals with known or suspected adherence challenges, a reduced dose of 160 mg once daily (QD) may represent a more pragmatic approach. Although higher exposure to lazertinib is associated with more toxicity [5], as it stands, it is unknown which lazertinib PK-parameter best predicts toxicity of treatment. Because the alternative dosing regimens in patients with the *GSTM1* null genotype all lead to lower exposure for the maximum, minimum and average concentration, we consider it likely that toxicity decreases in this subgroup of patients. A prospective evaluation of the toxicity profile of genotype-guided dosing of lazertinib in combination with amivantamab should provide solid evidence on the magnitude of toxicity reduction. In our analysis, we propose a 33–50% cumulative dose reduction in patients with the *GSTM1* null genotype. Since this dose reduction aligns with the label advising to perform a 33% dose reduction as a first step in toxicity management, we consider it likely that our alternative dosing regimens influence the toxicity profile of lazertinib. Since *GSTM1* genotype impacts the clearance of lazertinib by an approximate two-fold and the inter-individual variability in clearance is approximately 37% [5], this shows that the impact of *GSTM1* on drug exposure is not negligible and that genotyping can be used to individualize the dose.

According to the label, stepwise dose reductions are advised in case of toxicity, with a first reduction of 33% (from 240 to 160 mg) and a second reduction of 50% (from 160 to 80 mg) [5]. This stepwise strategy can be preserved with the every-other- day regimen, but is not applicable to the fixed 160 mg QD approach due to the limitation in tablet sizes of 80 and 240 mg. In such cases, one might consider administering 80 and 160 mg on alternating days as a first dose reduction.

The here proposed genotype-guided alternative dosing strategies are informed by pharmacokinetic modelling and simulation and have not yet been clinically tested. We argue that the available evidence underlying our simulations is sufficiently strong to implement these alternative dosing regimens in clinical practice. Given the high global prevalence of the *GSTM1* null genotype [8] and the substantial sample size of the population pharmacokinetic study conducted by the marketing authorization holder of lazertinib, the impact of *GSTM1* status on the pharmacokinetics of lazertinib is well-established. Therefore, the alternative dosing regimens proposed for *GSTM1* null individuals are highly unlikely to result in systemic exposures below those shown to be both clinically safe and effective in *GSTM1* non-null patients receiving the approved 240 mg QD dose.

Lastly, a clear relationship between systemic exposure and toxicity has been observed for lazertinib monotherapy in therapeutically relevant ranges [5]. Since lazertinib will be combined with EGFR inhibitor amivantamab, one might argue that direct translation of monotherapy findings to combination therapy is feasible. Nonetheless, we consider it likely that dose reductions of lazertinib in combination of with amivantamab will lead to less toxicity, as a dose reduction is already the first step in toxicity management according to the lazertinib label. Prospective evaluation *GSTM1*-guided dose individualization is needed to establish the magnitude of reduction of toxicity in *GSTM1* null patients on a reduced dose.

## Conclusion

In the era of precision oncology, overlooking *GSTM1* status in lazertinib dosing may no longer be justifiable. Genotype-guided dosing of lazertinib may overcome unnecessary toxicity and costs, while maintaining effective systemic exposure.

## Data Availability

No datasets were generated or analysed during the current study.
